# Microbial Community Associations With *Listeria monocytogenes* in Food Processing Environments: A Systematic Review and Meta‐Analysis

**DOI:** 10.1111/1541-4337.70277

**Published:** 2025-09-14

**Authors:** Jack Burnett, David Buckley, Dale A. Grinstead, Haley F. Oliver

**Affiliations:** ^1^ Department of Food Science Purdue University West Lafayette Indiana USA; ^2^ Diversey Fort Mill South Carolina USA; ^3^ Mountain Top Microbiology Highlands North Carolina USA

## Abstract

*Listeria monocytogenes* persistence in food processing environments challenges current understanding of microbial community dynamics. This systematic review and meta‐analysis examined peer‐reviewed studies that screened for *Listeria* spp. and performed culture‐independent metagenomics on FPE surface samples. Following PRISMA guidelines, we searched PubMed, Web of Science, and Food Science and Technology Abstracts databases, screening 464 studies, with 73 qualifying for full‐text review. Seven studies met the inclusion criteria for final analysis, encompassing 1659 environmental samples from meat processing (*n* = 4 studies) and produce facilities (*n* = 3 studies).

Meta‐analysis using random effects models revealed no significant correlation between *Listeria* presence and overall microbial community alpha diversity (Shannon: z = −0.89, *p* = 0.40; inverse Simpson and Chao1 indices similarly non‐significant). This finding challenges previous assumptions about the relationship between microbial diversity and pathogen persistence.

Differential abundance analyses identified three genera most frequently associated with *Listeria* presence across multiple studies: *Pseudomonas*, *Psychrobacter*, and *Acinetobacter*. These Gammaproteobacteria are characterized as aerobic biofilm formers capable of growth at refrigeration temperatures. One study using rigorous mixed‐effects modeling identified *Veillonella* as significantly associated with *L*. *monocytogenes* presence, suggesting potential anaerobic niche interactions within biofilm communities. Synthesis of metabolic capabilities reported in the literature suggests these associated genera may provide structural biofilm matrices and potentially complementary metabolic functions that could facilitate *L*. *monocytogenes* survival in FPE conditions. However, the genus‐level resolution of 16S rRNA amplicon sequencing data and methodological variations across studies limit definitive conclusions about specific metabolic interactions. These findings indicate that *L*. *monocytogenes* persistence appears to be associated with specific microbial partners rather than overall community diversity metrics. Understanding these ecological relationships may inform targeted control strategies focusing on biofilm‐forming genera that create favorable conditions for *Listeria* survival in food processing environments.

## 1 Introduction

*Listeria monocytogenes* persistence in food processing environments (FPEs, which for this review include production facilities, processing plants, and retail delis) poses significant public health risks (Scallan Walter et al. 2025; Palaiodimou et al. 2021; Stasiewicz et al. 2015). Persistence was recently defined by the European Food Safety Authority (EFSA) as “the ability of an organism to become established within FPEs and proliferate for long periods of time despite frequent sanitation efforts” (Koutsoumanis et al. 2024). This long‐term survival in FPEs is facilitated in part by the presence of biofilms, which provide protection against environmental stressors and sanitation (Y. Liu, Zhang, et al. 2016; Voglauer et al. 2025).

Extensive research into the traits of persisting *L*. monocytogenes isolates has occurred in hopes of identifying, then exploiting, the mechanisms that facilitate persistence (Camargo et al. 2019; Fagerlund et al. 2016; Lundén et al. 2003; Fox et al. 2011; Assisi et al. 2021). Various genetic features, clonal groupings, transcriptional regulation, *inlA* premature stop codons, and serotype were among the factors not sufficient, alone or together, to explain persistence in FPEs (Assisi et al. 2021; Daeschel et al. 2022; Doijad et al. 2015; Ferreira et al. 2014; Lee et al. 2019; Palaiodimou et al. 2021; Stasiewicz et al. 2015; Taylor and Stasiewicz 2019; Wang et al. 2015). Recent publications concluded that *L*. *monocytogenes* persistence is attributable to the microbial community and the complexity of their interactions within a biofilm matrix (Fagerlund et al. 2021; Luque‐Sastre et al. 2018; Taylor and Stasiewicz 2019; Voglauer et al. 2025).

In natural environments, *L*. *monocytogenes* may be found within complex microbial biofilms where it is vastly outnumbered by other bacteria (Zottola and Sasahara 1994; Fagerlund et al. 2021). *L*. *monocytogenes* are generally not considered strong biofilm formers (Y. Liu, Zhang, et al. 2016; Doijad et al. 2015; Lee et al. 2019; Wang et al. 2015; Janež et al. 2021) but can utilize the matrix of preexisting biofilms as an anchor for its own attachment and microcolony formation (Fagerlund et al. 2021; Puga et al. 2018; Pang and Yuk 2019). Laboratory experiments have demonstrated that, indeed, *L*. *monocytogenes* is able to colonize existing biofilms created by other bacterial species without altering biofilm composition or gene expression (Voglauer et al. 2025). This finding suggests that *L*. *monocytogenes* may behave more as a passive survivor and resident of biofilms rather than an active modifier, potentially explaining its ability to persist despite sanitation efforts.

Understanding biofilm formation in *Listeria*‐supportive communities is crucial for developing new prevention and control strategies (Giaouris et al. 2020; Fagerlund et al. 2017; Bai, Nakatsu, et al. 2021). Future development of biofilm prevention or removal chemistries requires the knowledge of (i) which biofilm‐producers co‐occur with *L*. *monocytogenes* and the structure and (ii) composition of the biofilm extracellular polymeric substances (EPS). Research into co‐occurrence patterns using culture‐based bacterial community profiling techniques has indicated that a variety of genera may interact with *L*. *monocytogenes* or *Listeria* spp. (Fox et al. 2014; Kocurek et al. 2024; N. T. Liu, Bauchan, et al. 2016; Schirmer et al. 2013; Zhao et al. 2004; Fagerlund et al. 2021; Callon et al. 2014; Rodríguez‐López et al. 2019). However, there is a paucity of discussion surrounding the metabolisms of the correlated genera and how those may compare to that of *Listeria* spp. The complexity of these relationships warrants dedicated discussion.

This systematic literature review (SLR) aims to identify the specific microbial taxa and metabolic interactions that facilitate *L*. *monocytogenes* persistence in FPEs. While the genus‐level resolution of 16S rRNA amplicon sequencing (16S) data imposes significant restrictions on such a discussion, it does not preclude the identification of metabolic features conserved at the genus level by those we are confident grow alongside *Listeria* spp.

Logically, *L*. *monocytogenes* growth within biofilms is a requirement for its persistence on nonfood contact surfaces (NFCS) to be of public health relevance. The long‐term detection of the same strain of *L*. *monocytogenes*, without growth, would require a high survival rate coupled with low rates of transfer to food contact surfaces (FCS) and products. Ultimately, such a scenario would not result in the long‐term outbreaks of *L*. *monocytogenes* that are of the highest public health concern (Koutsoumanis et al. 2024).

This review will compare extracted data from the included studies and contrast the metabolic capabilities known to be conserved among the relevant genera. Then we will compare those findings with what is known about the metabolic behavior of *L*. *monocytogenes* within mixed‐species biofilms. By addressing these gaps in the current research, this review aims to deepen our understanding of the microbial dynamics within FPEs, with a focus on biofilms and the metabolic interactions therein that, together, facilitate *L*. *monocytogenes* persistence.

## 2 Materials and Methods

The development of this SLR was guided by the Reporting Standard for Systematic Evidence Syntheses in Environmental Research (ROSES) checklist (Pussegoda et al. 2017) as well as the framework for SLRs in agriculture by Koutsos et al. (2019) based on Preferred Reporting Items for Systematic Reviews and Meta‐Analyses (PRISMA) (Koutsos et al. 2019; Liberati et al. 2009; Moher et al. 2009). These resources guided the screening processes, manuscript preparation, development of the research question, and search strategy. Their approach minimizes inherent bias found in many narrative reviews by ensuring that downstream discussion of references and any meta‐analyses are informed by an unbiased dataset.

### 2.1 Scoping and Planning

Scoping of the literature was performed via Google Scholar and PubMed to identify previous systematic reviews exploring the relationship between metagenomics and *L. monocytogenes* status in FPEs. Only the narrative review by Fagerlund et al. (2021) was returned. Research question formulation was a collaborative effort between the authors and industry stakeholders with guidance from Purdue University librarians.

The ubiquitous PICO (Population, Intervention, Comparator, Outcome) question formula has proven useful for clinical research but does not allow parsing by study contextual elements, which are key considerations in the present research (Booth et al. 2019). The PICo (Population, Phenomenon of Interest, Context) framework was deemed more appropriate. In our case, the (Stern et al. 2014) population is the microbial communities attached to NFCS within FPEs; the phenomenon of interest is the presence of *L. monocytogenes*; and the context represents the metagenomic profiling data of said communities.

### 2.2 Search for Relevant Studies

A list of search terms and Boolean operators was developed under the guidance of Purdue University librarians as part of a comprehensive reviews course during doctoral studies (Table S1). Iterations of the search strategy as it developed are provided in Table S2. Selected databases for the search were PubMed, Web of Science, and Food Science and Technology Abstracts (FSTA), as recommended by Purdue University librarians.

The search strategy incorporated both controlled vocabulary terms and free‐text keywords to maximize the retrieval of relevant studies. Medical Subject Headings (MeSH) were utilized in PubMed searches to capture articles indexed with standardized biomedical terminology. Equivalent controlled vocabulary approaches were applied where available: Web of Science searches relied on keyword and citation indexing, as this database does not employ subject headings for topic control, while FSTA searches utilized the database's proprietary food science terminology thesaurus. This multi‐faceted approach ensured coverage across different indexing systems and vocabularies used by each database.

Several potential sources of bias were considered and addressed in the search strategy design. Language bias represents a potential limitation of this review due to the restriction of searches to English‐language publications, which may have excluded relevant studies published in other languages. This restriction was implemented due to resource constraints and translation limitations. However, the effect of English‐language restriction on evidence synthesis is limited for most medical topics (Dobrescu et al. 2021). Database selection bias was minimized through the deliberate selection of three complementary databases with different coverage areas and indexing approaches. The selected combination provided coverage of biomedical literature (PubMed), multidisciplinary scientific literature (Web of Science), and specialized food science literature (FSTA). Previous studies have demonstrated that searching only one database can be insufficient to retrieve all references for systematic reviews, with single‐database searches missing a substantial proportion of relevant studies (Bramer et al. 2017; Lemeshow et al. 2005).

### 2.3 Abstract and Full‐Text Screening

The searches resulted in a list of citations, including abstracts, which were exported from each database and uploaded to the Covidence (Veritas Health Innovation Ltd., Melbourne, Australia) systematic review software program; the software performed de‐duplication. Screening was performed in two stages: an initial abstract followed by a full‐text screening.

Abstract screening was performed independently by two subject matter experts (SMEs). There was a single exclusion criterion at the abstract screening stage. Simply, if the study was clearly unrelated to *L. monocytogenes*, *Listeria* spp., or environmental microbiomes of any kind, it was excluded. Studies were included for full‐text review if they contained metagenomic data or any attempt to characterize the population distribution of a microbial community. Only one vote for inclusion was needed to qualify for full‐text screening.

**FIGURE 1:**
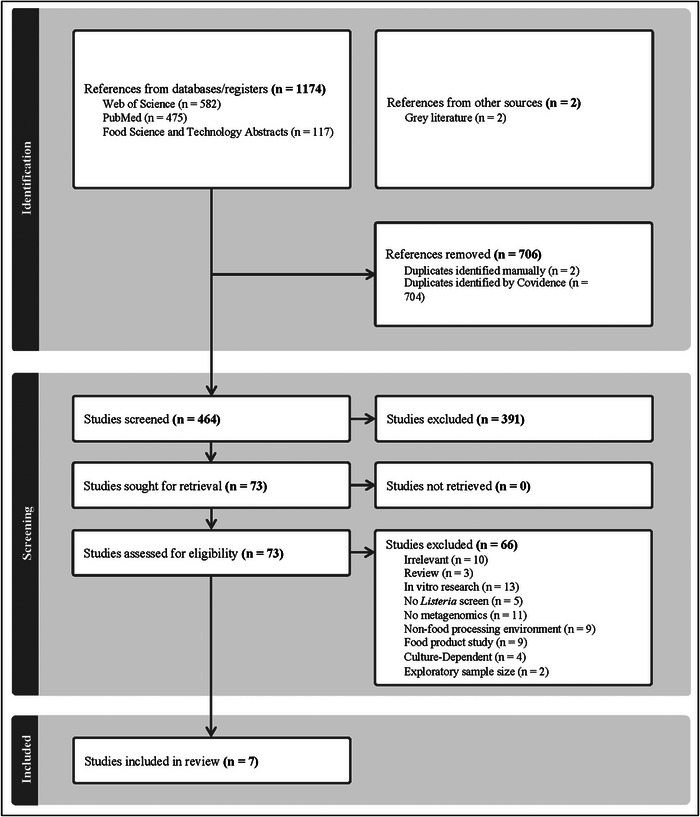
PRISMA Statement.

In a separate process, the same SMEs individually reviewed the resulting full texts. Excluded studies were binned by rationale. Conflicts between SMEs were to be resolved through discussion, but none arose. Exclusion and inclusion criteria and results are listed in Figure 1. The included studies are summarized in Table 1.

**TABLE 1:** Characteristics of studies included in systematic literature review of metagenomics and *Listeria* presence in food processing environments.

					Sampling approach					
Study name^a^	Country^b^	Environment^c^	Organism (s)^d^	Positive samples^e^	Sample type^g^	Swab strategy^f^	DNA extraction	Primersh	Platform^h^	Database^h^
Tan et al. (2019)	United States	Three tree fruit packing facilities; zone 3	*L. monocytogenes*	66/117 (56.4%)	Surface swabs	Adjacent samples	Dneasy PowerSoil Kit (Qiagen)	505F‐v2, 806R‐v2	Illumina miseq	Greengenes v13.8
Zwirzitz et al. (2021)	Austria	Large‐scale pork processing; six conveyors, PPE, and meat product surfaces	*Listeria* spp.	88/176 (50%)	Surface swab	Single sample	QiAamp DNA Stool Mini Kit (Qiagen)	27F, 1429R	Pacbio sequel	SILVA v138
			*L. monocytogenes*	24/176 (13.6%)						
Shedleur‐Bourguignon (2021)	Canada	Swine Slaughterhouse; Conveyors	*L. monocytogenes*	36/294 (12.2%)	Surface Wipe	Single Sample	Phenol‐Chloroform Protocol	515F, 806R	Illumina Miseq	SILVA v132
Belk et al. (2022)	United States	Small Meat Processing Facility (New Construction); Door Handles and Floor Drains	*Listeria* spp.	40/868 (4.6%)	Surface Swabs	Adjacent or Sequential Samples (unclear)	Dneasy PowerSoil Kit (Qiagen)	515F, 806R	Illumina Miseq	SILVA v138
			*L*. *monocytogenes*	26/868 (3.0%)						
Cherifi et al. (2022)	Canada	Swine Slaughterhouse; Conveyors	*L. monocytogenes*	13/48 (27.1%)	Surface Swab	Single Sample	FastPrep (MP Biomedical)	515F, 806R	Illumina Miseq	SILVA
Rolon (2023)	United States	Tree Fruit Packing Facilities; Zone 3	*L. monocytogenes*	81/107 (75.7%)	Surface Swabs	Adjacent Samples	Dneasy PowerSoil Kit (Qiagen)	505F, 806R	Illumina Miseq	SILVA v132
Townsend et al. (2023)	United States	18 Fresh Produce Distribution Centers; Zone 3, primarily Floors	*Listeria* spp.	18/303 (5.9%)	Surface Swabs	Adjacent Samples	Dneasy PowerSoil Pro Kit (Qiagen)	Custom for V3 and V4 regions	Illumina Miseq	Genome Taxonomy Database v95

^a^ the first author and year of publication.

^b^ country in which the study was conducted.

^c^ the facility type studied.

^d^ whether the study screened samples for *Listeria* spp., *L. monocytogenes*, or both.

^e^ prevalence of *L. monocytogenes* and/or *Listeria* spp.

^f^ swab strategy employed for sample collection.

^g^ type of sample collected.

^h^ sequencing methodology, including primers, platform, and reference database used.

### 2.4 Data Extraction From Included Studies

For each included study, the following data were systematically extracted when available: study location, facility type, sampling methodology, *Listeria* detection methods, sequencing platform and protocols, bioinformatics pipelines, statistical methods, and key findings regarding microbial diversity and differential abundance.

Whenever possible, each study's means and standard deviations of diversity metrics for *Listeria* spp. or *L*. *monocytogenes*‐positive and ‐negative samples were calculated directly from their raw data. Cherifi et al. (2022) provided said data in their supplementary materials, while the data from both Tan et al. (2019) and Rolon et al. (2023) were provided by their mutual corresponding author and are included in this review as Table S3.

The data from Townsend et al. (2023) was extracted from their manuscript's box‐and‐whisker plots that compared Shannon, Chao1, and inverse Simpson indices by *Listeria* spp. result. Belk et al. (2022) reported Shannon diversity by *Listeria* results at the room level in their supplementary data. For both studies, the means and standard deviations of the *Listeria*‐negative plots were calculated by first extracting five‐figure summaries (quartiles, minimum, and maximum) using *WebPlotDigitizer* v4.6 in manual mode (Rohatgi 2021), then estimating corresponding means and standard deviations using the R package *estmeansd* as previously described (McGrath et al. 2023; McGrath et al. 2020; Cai et al. 2021). This was appropriate for studies with a large number of negative samples; however, the limited number of positive samples meant the normal distribution assumption required for the *estmeansd* package was inappropriate (Luo et al. 2018). Fortunately, the limited number of *Listeria*‐positive datapoints meant they were individually discernible in the figures. *WebPlotDigitizer* v4.6 (Rohatgi 2021) was used to reconstruct the *Listeria*‐positive dataset, from which the mean and standard deviation were then directly calculated.

Belk et al. (2022)’s data required pooling of room‐level means and standard deviations. The overall facility means and standard deviations for negative samples were determined by averaging the weighted means and pooling their standard deviations via the *sdpool* function in the R package *effectsize* v0.8.6 (Ben‐Shachar et al. 2020). Shedleur‐Bourguignon et al. (2021) provided *p* values and the corresponding averages for diversity indices broken down by *Listeria* spp. Using *WebPlotDigitizer* v4.6 in manual mode (Rohatgi 2021), a standard deviation estimate was calculated based on the dot plots provided in the Supporting Information. Neither raw diversity data nor an alternative data extraction approach was available for Zwirzitz et al. (2021), so it was not included in the subsequent meta‐analysis.

### 2.5 Meta‐Analysis of Alpha Diversity Indices by *Listeria* Presence

Extracted data from the included studies (Table S4) were imported into R v4.2.3 for analysis. The R package *meta* v6.5‐0 (Balduzzi et al. 2019) and its *metacont* function were used to perform a random effects meta‐analysis of Shannon diversity indices between *Listeria*‐positive and ‐negative samples. Specifically, we deployed a DerSimonian–Laird random effects model with *τ* ^2^ estimated using the restricted maximum‐likelihood (REML) method. The *forest* function was used to generate a forest plot of the Shannon diversity meta‐analysis using default settings and random effects model output. Additional meta‐analyses were performed using inverse Simpson and Chao1 diversity indices where available. Heterogeneity was assessed using Cochran's *Q* test and quantified using *τ* ^2^ and *I* ^2^ statistics. Finally, since Tan et al. (2019) and Rolon et al. (2023) studied the same facilities, sensitivity analyses were conducted treating these as a single study to address potential dependence issues.

### 2.6 Differential Abundance Analysis Synthesis

For each included study, we systematically extracted information about statistical methods used for differential abundance analysis, including software packages, model specifications, and approaches for handling repeated measures or facility effects. We summarized the genera identified as significantly associated with *Listeria* presence across studies, noting methodological differences that might affect the interpretation of results.

The robustness of statistical approaches was evaluated based on their ability to account for study design features such as longitudinal sampling, spatial clustering, and multiple facility sampling. Studies employing mixed effects models or approaches specifically designed for microbiome data were distinguished from those using simpler approaches.

## 3 Results and Discussion

The primary objective of this systematic review was to elucidate the complex relationships between *L. monocytogenes* and the microbial communities within FPEs by comprehensively analyzing peer‐reviewed metagenomic studies. Our findings challenge the theory that microbial diversity directly correlates with *L*. *monocytogenes* prevalence. Instead, our findings support the notion that specific bacterial genera play critical roles in creating favorable conditions for *L. monocytogenes* within multispecies biofilms. The meta‐analysis revealed no significant correlation between *Listeria* presence and overall microbial diversity, emphasizing the need for more sophisticated approaches to understanding bacterial community dynamics in FPEs.

### 3.1 Search and Screening Process Results

The search and screening results are depicted in Figure 1. Web of Science, PubMed, and FSTA database searches resulted in 582, 475, and 117 publications, respectively. The Covidence de‐duplication algorithm removed 708 duplicates, including all 117 from the FSTA database. This resulted in 464 references being passed to the initial abstract screening (Table S5). Among these references, there were results unique to both Web of Science and PubMed.

Abstract screening resulted in the elimination of 391 references, resulting in 73 references remaining for the full‐text screening. All 73 references were retrieved via Purdue University Libraries (West Lafayette, IN, United States).

In the abstract screening phase, SMEs included any studies that they thought had *any* chance of containing relevant data. For that reason, 10 manuscripts removed during the full‐text review were binned as “irrelevant,” meaning they were among those long‐shot inclusions that didn't come close to panning out. There were 13 references that were excluded because they studied interactions between *Listeria* spp. and other bacteria in vitro. This was the most frequently cited exclusion criterion. There were 11 studies that were sampling for *Listeria* spp. in some fashion but didn't perform any metagenomics; this is in addition to four references that were excluded on account of the culture‐dependent nature of their metagenomic data. This is because culture‐based metagenomics is estimated to detect only 0.1% of the actual population in complex microbial communities (Cao et al. 2017).

Nine studies were excluded because they were food product microbiome studies, often fermentation‐related research. Nine more were excluded because they were studying communities outside of FPEs, such as wastewater, streams, and soil.

Five exclusions were studies of FPE microbiomes, but there wasn't a screening for *Listeria* spp. or *L*. *monocytogenes*. Three references were reviewed, whose relevant referenced studies were confirmed to be captured by our search and screening process. Finally, there were three studies that were excluded that met all inclusion criteria, but upon full‐text review, we found sample sizes to be too small to be held up against the more recent included references.

### 3.2 Notable Exclusions

This selection of excluded studies provides important context for understanding the methodological standards applied in this systematic review and highlights the evolution of metagenomic approaches in FPEs.

#### 3.2.1 Kocurek 2024 (FDA “Quasi‐Metagenomics”)

Kocurek et al. (2024) conducted a comprehensive study characterizing the microbial profile of *Listeria* spp.‐positive samples from FPEs. However, this study was excluded due to its culture‐dependent approach to microbiome characterization. The researchers performed 16S rRNA profiling only after pre‐enrichment for *Listeria* spp., which fundamentally alters the microbial community composition compared to direct environmental sampling. This methodological choice was driven by practical constraints rather than scientific preference. As an FDA study, samples were collected as part of routine regulatory operations with strict chain‐of‐custody requirements that restricted when additional material could be collected for microbiome analysis. The authors acknowledged this limitation and termed their approach “quasi‐metagenomics” to distinguish it from true culture‐independent methods.

#### 3.2.2 Dziecol 2016 (Insufficient Sample Size)

Dzieciol et al. (2016) provided essential preliminary data that helped justify the larger, more robust studies included in this review. The researchers characterized 16S rRNA amplicon profiles of both biofilm swabs and drain water samples from four floor drains within a cheese processing facility with historically high *Listeria* spp. Prevalence. Only one of the four biofilm samples was positive for *Listeria* spp., resulting in a sample size too small for statistical analysis.

Despite this limitation, the study made several important contributions. The researchers astutely differentiated between drain biofilm communities (representing established sessile populations) and drain water communities (representing transient planktonic populations), finding markedly different metagenomic profiles between these two sample types from the same drains. This observation presaged findings from more recent studies showing disagreement between swab and biomass samples from retail deli drains (Britton et al. 2023).

#### 3.2.3 Liu 2016 (Culture‐Dependent Community Profiling)

Y. Liu, Zhang, et al. (2016) characterized microbial communities from 100 drain sponge samples collected across five food processing facilities in China. *L. monocytogenes* was detected in eight samples from two facilities. Only the eight positive samples underwent subsequent microbial community characterization using three culture‐dependent molecular approaches: clone libraries, terminal restriction fragment length polymorphism (T‐RFLP), and (DGGE). The study design, while appropriate for its era, represents an intermediate approach between purely culture‐based methods and modern culture‐independent NGS techniques.

Despite these limitations, the study provided critical early insights into biofilm ecology that foreshadowed the current understanding of *L. monocytogenes* environmental persistence. The researchers identified dominant bacterial phyla in *L. monocytogenes*‐positive drains and crucially hypothesized that *L. monocytogenes* represents a minor component of biofilm communities, relying on EPS produced by other microorganisms for protection against environmental stressors. This biofilm matrix dependency hypothesis remains widely accepted in the literature, though definitive experimental proof of *L. monocytogenes* reliance on heterologous EPS for environmental persistence has not yet been established. The study's contribution to the theoretical understanding of *L. monocytogenes* ecology was substantial, even though its methodological approach prevented inclusion in this review.

#### 3.2.4 Rodriguez‐Lopez 2019/2020 (Exploratory Studies)

Two exploratory studies by Rodríguez‐López et al. (2019, 2020) were excluded due to limited sample sizes for metagenomic sequencing, despite screening larger numbers of environmental samples. In their 2019 study, the researchers screened 40 *L. monocytogenes*‐positive samples but selected just three for 16S sequencing. Their subsequent 2020 study expanded this to nine *L. monocytogenes*‐positive biofilm samples for sequencing. These studies provided crucial proof‐of‐concept data that demonstrated the feasibility of culture‐independent approaches in FPEs.

The progression from these exploratory studies to the comprehensive investigations included in our analysis illustrates the rapid advancement of metagenomic approaches in food safety research. The preliminary data from Rodriguez‐Lopez et al. helped establish the scientific rationale and methodological frameworks that enabled subsequent researchers to design adequately powered studies capable of detecting genuine ecological associations.

### 3.3 Overview of Included Studies

#### 3.3.1 Tan 2019

This foundational research examined the composition of microbiota in three tree fruit processing facilities in the Northeastern United States. The study employed 16S amplicon sequencing using Illumina MiSeq technology combined with enrichment‐based screening for *L. monocytogenes*. A total of 117 samples were collected from “zone 3” surfaces, specifically floor swabs along conveyor processing systems, with each of three facilities contributing 39 samples. The *L. monocytogenes* prevalence varied dramatically between facilities: facility 2 (F2) had 100% positive samples (39/39), while facilities 1 and 3 had 28% and 41% positive rates, respectively. This extreme difference in contamination patterns proved both illuminating and problematic for downstream statistical analysis.

Diversity metrics were reported at the facility level, and pairwise comparisons revealed that F2 clustered separately from the other facilities on principal coordinate analysis plots, with significantly different alpha diversity compared to the other two facilities. However, the statistical approach presented limitations for broader inference. While the authors calculated diversity differences by facility, this approach reduced their effective sample size to three facilities rather than 117 individual samples.

Rolon et al. (2023) subsequently performed differential abundance analysis on this dataset using ALDEx2 v1.26.0. When considering the Tan et al. (2019) data alone, 34 amplicon sequence variants (ASVs) from *Pseudomonas* and two from *Stenotrophomonas* were significantly higher in *L. monocytogenes*‐positive samples, while 22 ASVs from *Acinetobacter* were significantly higher in negative samples. Importantly, when F2 was excluded from analysis due to its 100% positive rate, no significant differential abundances were detected, highlighting the influence of this extreme contamination pattern on the results.

##### 3.3.1.1 Schedleur‐Bourguignon 2021

This study represents one of the most methodologically rigorous approaches to differential abundance analysis in the reviewed literature. Researchers sampled beginning, middle, and end surfaces of six conveyors in a Canadian swine slaughterhouse, collecting 300 samples over multiple time points. Of these, 36 samples (12.2%) were positive for *L. monocytogenes*, with positives restricted to only three of the six conveyors handling half carcasses, Boston, and picnic cuts, while conveyors processing belly, loin, and ham cuts yielded no positives.

The statistical approach employed distinguished this study from others in the review. The researchers used MaAsLin v1 (Morgan et al. 2012), which specifically addresses the analytical challenges inherent in longitudinal microbiome studies. This approach employs a boosted permutational method to compare each operational taxonomic unit (OTU) against metadata variables, then applies mixed models to test relationships while properly accounting for dataset autocorrelations introduced by repeated measurements both longitudinally and by conveyor. This rigorous statistical framework led to the identification of only *Veillonella* spp. as significantly associated with *L. monocytogenes* presence, a finding that contrasts with the larger numbers of associated genera identified in studies using less appropriate statistical models. The conservative nature of this result likely reflects proper control for repeated measures, which many other studies failed to implement.

##### 3.3.1.2 Zwirzitz 2021

This comprehensive study followed pork cuts through their processing flow in an Austrian facility, sampling not only meat surfaces but also employee personal protective equipment (PPE) and nearby FCS and NFCS. The design provided unique insights into cross‐contamination dynamics, with 176 samples collected, representing 88 meat surfaces, 48 FCS, 32 PPE surfaces, and 8 NFCS. *Listeria* spp. were detected in exactly half of all samples (88/176), with 13.6% positive for *L. monocytogenes*. Exactly half (88/176) of all samples tested positive for *Listeria* spp., and 13.6% (24/176) were positive for *L. monocytogenes*. According to their PERMANOVA output, the variables for sample type (environmental vs. product) as well as position (sampling location) and *Listeria* status were significant factors in “determining the structure of the microbial communities.” It is unclear which metrics they used for their statistical analysis, nor do they share any additional information gleaned from post hoc testing. Alpha diversity metrics were only reported for meat surface samples, precluding them from being included in our Shannon index meta‐analysis. Only beta diversity results were presented for environmental swabs.

There were several genera determined to be linked to the *Listeria* status of the sample; considering only environmental surface samples, *Acinetobacter*, *Janthinobacterium*, *Brachybacterium*, *Carnobacterium*, *Chryseobacterium*, *Moraxella*, and *Anoxybacillus* all had abundances that correlated positively with *Listeria* presence. However, the statistical analysis presented methodological concerns. The researchers employed Spearman rank correlations and DESeq2 for differential abundance analysis without accounting for the longitudinal nature of their sampling design.

There are two notable, well‐supported conclusions from this study. First, they demonstrated that contamination by *Listeria* spp. occurred during processing. *Listeria* spp. weren't found on carcass surfaces prior to processing, nor on the FCS of the first processing step. Yet, in the downstream meat cutting and packaging areas, *Listeria* spp. were found on meat surfaces. This strongly suggests environmental cross‐contamination. Second, the overall microbial profile on the meat surfaces quickly changes to reflect the dominant taxa found in the facility's core microbiome. A decrease in species richness on meat surfaces when comparing samples from the beginning and end of the processing flow coincided with a clear shift in the community composition based on beta diversity analysis. Taken together, these support the authors’ conclusion.

##### 3.3.1.3 Belk 2022

This longitudinal study provided unique insights by following a newly constructed meat processing facility from the first post‐construction cleaning through 18 months of operation. Samples were collected monthly from floor drains and door touch points, yielding 868 total samples with 4.6% positive for *Listeria* spp. and 3.0% positive for *L. monocytogenes*. After 14 sampling events, the researchers state they had collected 630 drain swabs and 300 samples of door touch points. The authors state that 4.6% of the samples (40/868) contained *Listeria* spp. Of the 40 positive samples, 26 were determined to be *L*. *monocytogenes*. The most positive samples were found in the live animal holding and harvest spaces, but positive swabs were taken in all parts of the facility.

The facility's design and sampling approach revealed important spatial patterns. Live animal holding and harvest areas clustered separately on PCoA plots from processing areas, with the former dominated by *L. innocua* and the latter by *L. monocytogenes*. The authors appropriately attributed this pattern to environmental differences (temperature, sanitation intensity, and soil introduction) rather than microbial competition.

Methodologically, the study employed a Random Forest model and PERMANOVA for differential abundance analysis. However, like Zwirzitz et al. (2021), the analysis did not specifically control for repeated measures in the statistical models. The study identified *Acinetobacter*, *Chryseobacterium*, *Psychrobacter*, and *Flavobacterium* as associated with *Listeria* spp. presence. Importantly, the authors found no significant alpha diversity differences between *Listeria*‐positive and negative samples when analyzed by room function, though a mixed model approach incorporating room as a random effect would have provided more robust inference.

##### 3.3.1.4 Cherifi 2022

This study took a network analysis approach to examine post‐sanitation surfaces in a Canadian swine slaughterhouse cutting room. Researchers collected samples from three locations along each of three conveyor belts during four sampling visits over 6 months, yielding 48 total samples, with 13 (27.1%) positive for *L. monocytogenes*. Their temporal analysis revealed dynamic community composition, with *Pseudomonas* dominating the first sampling visit, *Acinetobacter* dominating visits two and three, and *Sphingomonas* becoming dominant in the final sampling. Interestingly, only visits two and three differed significantly in pairwise PERMANOVA comparisons, suggesting that *Acinetobacter* dominance may have represented a transitional state.

The network analysis approach yielded notably different results from other studies. After applying correlation thresholds and filtering for direct relationships with *Listeria*, no genera showed positive correlations with *L. monocytogenes* presence. Instead, six genera showed negative correlations: *Herminiimonas*, *Bryobacter*, *Caulobacter*, *Sphingomonas*, *Mycobacterium*, and an unidentified member of Elusimicrobia.

##### 3.3.1.5 Townsend 2023

This comprehensive study enrolled 18 produce distribution centers across the United States, with an average of 17 samples per location (303 total samples). The sampling focused on zone 3 surfaces, including floors (*n* = 175), cleaning‐related surfaces (*n* = 84), barriers (*n* = 38), and walls (*n* = 6). Only 5.9% (18/303) of samples were found to contain *Listeria* spp. via standard enrichment screening. The authors report that 89.4% of samples (271/303) were from dry surfaces. The previously established association between standing and leaking water and Listeria prevalence (Estrada et al. 2020; Burnett et al. 2022; Simmons et al. 2014), in addition to the highly hydrophilic surfactome of *Listeria* spp. helps explain the relatively low prevalence found on zone 3 surfaces when compared to prior studies of similar (Burnett et al. 2020; Townsend et al. 2021; Sullivan and Wiedmann 2020; Estrada et al. 2020). It is also noteworthy that 71.6% (217/303) of the samples collected were from refrigerated spaces.

The statistical approach was exemplary for a cross‐sectional study design. The researchers employed both DESeq2 (Love et al. 2014) and ANCOM‐BC (Lin and Peddada 2020) for differential abundance analysis, with ANCOM‐BC specifically controlling for sampling fraction bias. The newer ANCOM‐BC method identified different results from DESeq2, highlighting the importance of method selection in microbiome analysis.

Alpha diversity analysis using Wilcoxon rank sum tests with continuity correction found significantly lower Shannon, Chao1, and observed diversity values in *Listeria*‐positive samples. Despite the authors' concerns about small sample size effects (18 positive vs. 285 negative), the appropriate statistical test was performed with small‐sample corrections applied. Beta diversity was assessed via PCoA plots, and two distinct clusters were present. However, the authors state that none of the metadata variables or groupings explored in their analysis corresponded to these clusters. A PERMANOVA analysis of the remaining factors indicated that many variables were significant but had low *R* ^2^ values, meaning that most of the separation in the clustering was still unexplained (Bruce and Bruce 2017). Season (*p* < 0.01; *R* ^2^ = 0.069) was the most influential factor, explaining just 6.9% of the variation in distance. Neither sequencing batch effects nor DNA concentration ranges at any step of the 16S amplicon library preparation accounted for the clustering. The unexplained clusters are intriguing; these researchers were very thorough in their collection and analysis of metadata variables, and none of them could explain the clustering.

Differential abundance analysis identified *Psychrobacter*, *Pseudomonas*_E, and *Carnobacterium* as significantly increased in *Listeria*‐positive samples. The identification of different *Pseudomonas*_E ASVs in both positive and negative samples illustrates the species‐ and strain‐level variation in interactions with *Listeria*, supporting laboratory findings that these relationships depend on specific bacterial strains and environmental conditions.

##### 3.3.1.6 Rolon 2023

This follow‐up study to Tan et al. (2019) revisited the same three Northeastern tree fruit facilities, providing valuable temporal validation and methodological advancement. The study employed both 16S amplicon sequencing for bacterial communities and ITS2 sequencing for fungal communities, along with exploratory shotgun metagenomics on three samples that were positive for *L*. *monocytogenes*. F2 maintained its 100% *L. monocytogenes* positive rate in Year 2, while facilities 1 and 3 showed increased positivity rates, with the increase in facility 3 reaching statistical significance. This consistency in contamination patterns across years strengthened the ecological interpretations from the original study.

The differential abundance analysis employed ALDex2 v1.26.0 (Nixon et al. 2024; Gloor et al. 2016; Fernandes et al. 2013; Fernandes et al. 2014), which treats microbiome data as compositional rather than count data. 16S results were compared between *L*. *monocytogenes* positive and negative samples, considering each year's data individually as well as combined. No ASVs significantly differed in relative abundance when combining the 2 years. When considering Y1 alone, 34 ASVs from the genus *Pseudomonas*, and two from *Stenotrophomonas* were significantly higher in abundance in *L*. *monocytogenes*‐positive samples, while 22 ASVs from the *Acinetobacter* genus were significantly higher in abundance in *L*. *monocytogenes*‐negative samples. There were no significant differences when examining Y2's data via ALDex2. However, a random forest model found two genera among the 30 ASVs most informative for classification, which also had increased mean relative abundances in *L*. *monocytogenes*‐positive samples: *Pseudomonas* and *Microbacterium*.

The authors conclude that their data is biased due to F2's rate of positive samples. Examination of the authors' GitHub code revealed they used the aldex.ttest function rather than aldex.glm, which would have been necessary to properly account for repeated measures within and between facilities and years. The authors are correct; this methodological limitation likely affected the interpretation of the differential abundance results, particularly given F2's extreme contamination pattern.

The shotgun metagenomics component provided unprecedented insights into *L. monocytogenes* abundance in environmental samples. Culture‐independent detection showed *L. monocytogenes* comprising 0.002%–0.007% of bacterial reads in positive samples, confirming long‐standing observations that this pathogen represents a minor fraction of total bacterial populations where present. Additionally, *Listeria* spp. other than *L. monocytogenes were* detected in all three samples, providing important data on the utility of *Listeria* spp. as indicator organisms. The species‐level resolution of shotgun sequencing revealed that *Pseudomonas fluorescens* and *P. rhizospharae* dominated facilities 1 and 2, with additional species including *P. koreensis*, *P. putida*, *P. syringae*, *P. chlororaphis*, and *P. resinovorans* also present. This taxonomic detail supports the hypothesis that specific *Pseudomonas* species, rather than the genus, drive associations with *L. monocytogenes*.

### 3.4 Meta‐Analysis of Microbial Diversity and *Listeria* Presence

**FIGURE 2:**
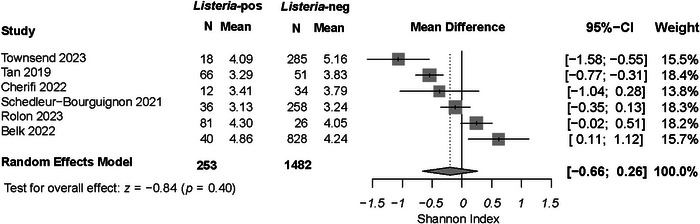
Forest plot of meta‐analysis results comparing the Shannon index of diversity for *Listeria*‐positive and ‐negative samples within each study.

Results of the meta‐analysis are displayed in a forest plot (Figure 2). A test for heterogeneity was significant (*τ* ^2^; *p* < 0.01), indicating that there is no agreement across the studies, and therefore, a random effects model was appropriate for a test of the overall effect (Bruce and Bruce 2017). The results indicate a lack of evidence that the presence of *Listeria* spp. is correlated to Shannon diversity (*z*; *p* > 0.05). To further investigate this result, we repeated this meta‐analysis using a subset of the publications that reported inverse Simpson and/or Chao1 diversity matrices. These additional analyses found significant differences between *Listeria* spp.‐positive and ‐negative samples (*z*; *p* > 0.05). Shannon diversity measures the richness of the community, meaning that it quantifies the number of taxonomical units present in a sample (Nagendra 2002), while the inverse Simpson index measures the evenness of the taxa within a community; it quantifies the distribution of abundances amongst the groups present (Buffalo 2015). Our results indicate that neither the number of species nor their distribution is directly correlated to the presence of *L*. *monocytogenes* or *Listeria* spp.

This result differs from prior research. For instance, Tan et al. (2019) found that in F2, the rarefaction curves and alpha diversity matrices both indicated significantly lower Shannon and inverse Simpson diversity matrices when compared with the two other facilities in the study. The authors interpret this as support for earlier suggestions that lesser nutrient competition and increased bacteriocin production in communities with lower diversity could facilitate the persistence of *L*. *monocytogenes* (Leriche and Carpentier 2000). Other research has similarly suggested that higher diversity could be a barrier to growth and persistence of *L*. *monocytogenes* (Retureau et al. 2010; Vivant et al. 2013), and one “notable exclusion” study also observed the potential for a relationship between *Listeria* spp. presence and community diversity (Rodríguez‐López et al. 2019).

Alternatively, Belk et al. (2022) found no significant difference in alpha diversity metrics between samples with and without *Listeria* spp. When considering only the processing areas, the average diversity in this study was *higher* in *Listeria* spp.‐positive samples. The authors note their surprise at this result in the context of Tan et al. (2019)’s findings and propose that the source of the inconsistency may be due to the high levels of diversity that were found on their sampling sites immediately following the post‐construction cleaning, which later stabilized at lower levels. Townsend et al. (2023) was the only other study that found significantly lower alpha diversity values for *Listeria* spp. positive samples. However, the authors were skeptical of the inferential power of these results due to their low number of positive samples (18 positive, 285 negative). They point to the overlap of their *Listeria* spp.‐positive and ‐negative alpha diversity box plots as evidence that an increased sample size might change the results of their analysis. Indeed, the Shannon diversity metric has been repeatedly suggested to be biased in small sample sizes (Konopiński 2020).

A second iteration of the meta‐analysis was carried out in which facility type (produce vs. meat) was added as a subgroup variable, forming two blocks of three studies. The results of this test were also insignificant (*z*; *p* > 0.05). Additionally, since Tan et al. (2019) and Rolon et al. (2023) both studied the same three facilities, the assumption of their independence in the random effects model cannot be made. As such, both the overall and facility‐type subgroup meta‐analyses were repeated, wherein those two studies were treated as a single study. These changes also did not alter the outcome of the meta‐analyses. These sensitivity analyses should not be interpreted as evidence that facility type is an *insignificant factor*; rather, this iteration of the meta‐analysis provides confidence that facility type is unlikely to be a *confounding factor* in this specific model.

This meta‐analysis demonstrates a lack of evidence that the presence or absence of *L*. *monocytogenes* or *Listeria* spp. on an FPE surface sample is correlated with any of the broad community structure metrics. The consistent presence of certain aerobic biofilm formers in *Listeria*‐positive samples, combined with the notable absence of others, like *Bacillus*, *Staphylococcus*, and *Streptococcus*, suggests a nonrandom ecological pattern. The said pattern of associations and non‐associations that emerges from the included studies suggests that *L*. *monocytogenes* persistence in FPEs may depend on a balance between cooperative partners that provide structural support and metabolic benefits while avoiding strongly antagonistic species.

### 3.5 Differential Abundance Analyses

Table 2 outlines the statistical software deployed for differential abundance analysis, revealing the variety of statistical methodologies, experimental designs, and model selection across the studies reviewed. Notably, two studies, Shedleur‐Bourguignon et al. (2021) and Townsend et al. (2023), demonstrated exemplary data fitting with robust models. The former employed the R package *MaAsLin* v1, which differentiates microbiomes across metadata variables through a boosted permutational approach (Morgan et al. 2012). It subsequently applies a mixed model to test relationships while addressing the dataset's autocorrelations introduced by repeated measurements both longitudinally and by conveyor. Townsend et al. (2023) did not have a longitudinal element to their study.

**TABLE 2:** Summary of differential abundance analysis methods and associated bacterial genera across multiple studies.

	Differential abundance		
Study name^a^	Statistical approach^b^	Positively associated^c^	Negatively associated^d^
Tan et al. (2019)	ALDeX2 v1.26.0	None	None
	Random Forest	*Pseudomonas, Stenotrophomonas*	*Acinetobacter*
Zwirzitz et al. (2021)	Spearman Rank Correlations	*Janthinobacterium, Acinetobacter, Soonwooa, Anoxybacillus, Flavobacterium, Chryseobacterium, Moraxella, Arthrobacter, Brevibacterium, Brachybacterium, Carnobacterium, Serratia, Lelliottia, Halomonas, Staphylococcus, Rahnella1, Jeotgalicoccus, Rothia, Psychrobacter, Escherichia/Shigella, Glutamicibacter, Sphihngobacterium, Verticiella, Vitreoscilla, Mycetocola, Enterococcus, Avidovorax, Myroides, Paeniglutamicibacter, Brochothrix*	*Anaerobacillus, Ochrobactrum, Delftia*
	DESeq2	*Pseudomonas, Psychrobacter, Chryseobacterium, Acinetobacter, Arthrobacter, Brochothrix*	*Psychrobacter, Anaerobacillus*
Shedleur‐Bourguignon (2021)	MaAsLin v1	*Veillonella*	None
Belk et al. (2022)	Pearson's Chi‐squared	*Psyrchrobacter, Acinetobacter, Chryseobacterium*	None or Not Reported
Cherifi et al. (2022)	Network Analysis	None	*Herminiimonas, Bryobacter, Caulobacter, Sphingomonas, Mycobacterium*
Rolon (2023)	ALDeX2 v1.26.0	None	None
	Random Forest	*Pseudomonas, Microbacterium*	None or Not Reported
Townsend et al. (2023)	ANCOM‐BC (Log_2_ fold changes >1 or <‐1)	*Psychrobacter, Carnobacterium*	177 ASVs
	DESeq2	*Psychrobacter, Pseudomonas*	293 ASVs

^a^ Study name and publication year.

^b^ Statistical method or software package used for differential abundance analysis.

^c^ Bacterial genera showing positive association with *Listeria* presence.

^d^ Bacterial genera showing negative association with *Listeria* presence.

The remaining studies frequently used multiple statistical approaches, including permutational methods like PERMANOVA or bootstrapping. Unfortunately, it is not possible to permute the effect of longitudinal sampling; stratification is required to constrain the permutations to within the levels of the random variable. This common oversight suggests potential model selection biases necessitating a critical reassessment of conclusions.

For example, without accounting for the repeated measures, two samples would be treated as independent even if they were taken from the same exact surface at two different timepoints. In this situation, the dominant genera in those two samples are likely to have some overlap. Therefore, we would overestimate the significance of their abundance should both samples return the same *Listeria* result. Taking these methodological limitations into consideration, these models can be more accurately interpreted as having identified the dominant genera in the *Listeria*‐positive samples rather than establishing direct relationships or interactions. Despite these limitations, the consistent identification of similar taxa across studies using different methodologies suggests genuine ecological associations worth exploring further.

#### 3.5.1 Aerobic Metabolism and Biofilm Formation: Foundational Support for *Listeria* Persistence

Table 2 delineates each study's differential abundance results by *Listeria* spp. result. Identifying patterns among taxa whose abundance correlates with *L*. *monocytogenes* presence may unveil metabolic strategies and microcolony formation processes within biofilms, contributing to this pathogen's persistence. Notably, *Pseudomonas*, *Psychrobacter*, and *Acinetobacter* were identified by more than one study as significantly increased in abundance in *L*. *monocytogenes*‐ or *Listeria* spp.‐positive samples.

Four of the included studies identified *Pseudomonas* spp. as positively correlated in abundance to *L*. *monocytogenes* or *Listeria* spp. presence. It was also the dominant genus in *L*. *monocytogenes*‐positive samples in Cherifi et al. (2022) but was not differentially abundant. *Pseudomonas* spp. are recognized for creating porous, open‐architecture biofilms optimized for nutrient and waste transport (Bai, Liu, et al. 2021). The genus predominates in FPEs regardless of food commodity type (Fagerlund et al. 2017; Mertz et al. 2015; Xu et al. 2023), suggesting a complex coexistence with *L*. *monocytogenes* in multispecies biofilms (Fagerlund et al. 2017; Heir et al. 2018; Papaioannou et al. 2018; Pang et al. 2019; Puga et al. 2018).

Three of the studies included in this SLR found evidence that *Psychrobacter* spp. was significantly higher in abundance alongside *Listeria* spp. *Psychrobacter* spp. are frequently found among the predominant taxa of FPE microbial communities regardless of the food facility type and estimated associated nutrient flux (Xu et al. 2023). Additionally, exploratory metagenomic studies of floor drains in meat, cheese, and seafood FPEs found *Psychrobacter* spp. to be among the most abundant taxa in *L*. *monocytogenes*‐positive samples (Rodríguez‐López et al. 2019; Rodríguez‐López et al. 2020), supporting an earlier finding of the same (Fox et al. 2014).

*Acinetobacter* spp. were identified as increasingly abundant in *L*. *monocytogenes*‐positive samples in two reviewed studies. The robust biofilms they form, including in low‐temperature environments, are postulated to be the reason for their frequent survival of sanitation (Langsrud et al. 2016). A survey of 75 *Acinetobacter* spp. isolates determined 68% could form biofilms and 40% were strong EPS producers (Bala et al. 2016).

Pseudomonas, Psychrobacter, and *Acinetobacter* are all strictly aerobic organisms with robust biofilm‐forming capabilities, even under low temperatures (Langsrud et al. 2016; Mann and Wozniak 2012). The consistent association across studies suggests this is not coincidental but rather a fundamental ecological strategy employed by *Listeria* in FPEs. These aerobic biofilm formers likely create structured habitats that may provide *L. monocytogenes* with protected microenvironments conducive to its persistence.

#### 3.5.2 Biofilm Composition and Structure

The composition and architecture of biofilms formed by *Listeria*‐associated genera may reveal important mechanisms that facilitate *L. monocytogenes* persistence. The EPS produced by these genera not only form protective matrices but may also create microniches with specific physicochemical properties that support *Listeria* colonization and survival.

##### 3.5.2.1 Pseudomonas

*Pseudomonas* biofilms demonstrate remarkable diversity in extracellular matrix composition that may contribute to the observed species‐specific associations with *L. monocytogenes*. Analysis of EPS component distribution across the *P. fluorescens* group reveals that the species identified by Rolon et al. (2023) and shotgun sequencing (*P. fluorescens* and *P. rhizospharae) possess* distinct but complementary biofilm‐forming capabilities that could facilitate *Listeria* colonization (Heredia‐Ponce et al. 2021; Blanco‐Romero et al. 2020). *P. fluorescens*, which dominated the samples from facilities 1 and 2 in the shotgun analysis, retains multiple polysaccharide production systems, including Pel, poly‐β‐1,6‐*N*‐acetylglucosamine (PNAG), and alginate. This combination creates biofilms with robust structural matrices and high cation‐binding capacity through Pel's crosslinking with extracellular DNA (eDNA) (Harmsen et al. 2010; Heredia‐Ponce et al. 2021; Jennings et al. 2015). Since eDNA is thought to be important in *L. monocytogenes* biofilm formation (Jennings et al. 2015; Lee et al. 2019; Colagiorgi et al. 2016), *Listeria*‐derived eDNA could readily integrate into these Pel‐based matrices.

Studies of *Pseudomonas* biofilms reveal stark c‐di‐GMP gradients, with outer biofilm layers exhibiting high concentrations of this signaling molecule (Nair et al. 2017). In *P. aeruginosa*, c‐di‐GMP regulates production of multiple EPS components, including Psl polysaccharide, which forms fiber‐like structures essential for biofilm architecture. Crucially, c‐di‐GMP also induces sessile behavior in *L. monocytogenes* (Köseoğlu et al. 2015), suggesting that *Pseudomonas* biofilms provide chemical cues that promote *Listeria* biofilm formation and integration into established matrices.

Particularly interesting is the commonality of PNAG production across *Listeria*‐associated *Pseudomonas* species. The species Rolon et al. identified in *L. monocytogenes*‐positive samples using shotgun sequencing (*P. koreensis*, *P. putida*, *P. syringae*, *P. chlororaphis*, and *P. resinovorans*) all maintain PNAG biosynthetic capacity, supporting the notion that this polysaccharide could play a role in creating favorable conditions for *L. monocytogenes* persistence.

##### 3.5.2.2 Psychrobacter

*Psychrobacter* spp. biofilms demonstrate sophisticated molecular adaptations for cold environments that directly facilitate *L. monocytogenes’* persistence in refrigerated food processing facilities. These psychrotolerant bacteria produce highly specialized EPSs featuring novel polysaccharide structures and massive adhesin proteins that create exceptionally stable biofilm matrices under low‐temperature conditions (Hinsa‐Leasure et al. 2013).

*Psychrobacter* spp. biofilm formation is mediated by exceptionally large adhesin proteins that maintain functionality across the entire bacterial growth temperature range. The Cold Attachment Protein 1 (Cat1) from *P. arcticus* represents one of the largest characterized bacterial adhesins at 6715 amino acids, facilitating robust surface attachment and biofilm development at temperatures as low as 4°C (Koh et al. 2017). This cold‐adapted attachment mechanism enables stable biofilm establishment under refrigeration conditions where *L. monocytogenes* persistence is most problematic. Within biofilms, extracellular protein content increases dramatically under cold conditions (4°C), comprising 71%–77% of total EPS compared to minimal levels under mesophilic conditions. This temperature‐responsive matrix composition creates enhanced structural integrity and protective microenvironments that could shield associated *L. monocytogenes* from environmental stressors.

##### 3.5.2.3 Acinetobacter

*Acinetobacter* spp. biofilms exhibit sophisticated iron‐responsive regulatory systems that influence both matrix composition and potential interactions with *L. monocytogenes*. The major EPS components include PNAG and alginate, with production levels significantly influenced by iron availability (Modarresi et al. 2015). PNAG represents the predominant polysaccharide component in *A. baumannii* biofilms (Choi et al. 2009). This polysaccharide forms the structural backbone of their biofilms and co‐localizes with Csu pili to create hydrophobic biofilm patches (Pakharukova et al. 2018).

Biofilm‐associated protein (Bap) expression in *Acinetobacter* is highly regulated by iron availability, with biofilm formation enhanced under iron‐limited conditions (Azizi et al. 2016). This protein creates multidimensional biofilm architectures with internal water channels, providing structured habitats that could accommodate secondary colonizers like *L. monocytogenes* (Loehfelm et al. 2008). The iron‐responsive nature of *Acinetobacter* biofilm formation may explain the context‐dependent associations observed with *L. monocytogenes*. In iron‐rich environments, reduced biofilm formation could limit protective niches, while iron limitation enhances matrix production and potentially creates more favorable conditions for *Listeria* persistence.

##### 3.5.2.4 Common Matrix Features

The biofilm matrices produced by *Listeria*‐associated genera share critical structural features that create universally favorable conditions for *L. monocytogenes* colonization. Rather than representing specific co‐evolutionary partnerships, these genera independently produce biofilm architectures with compatible properties that facilitate passive *Listeria* integration.

The most intriguing commonality across *Listeria*‐associated biofilms is PNAG production. This polysaccharide appears consistently in biofilms from both *Pseudomonas* species and *Acinetobacter* species (Gedefie et al. 2021). PNAG's mechanical stability, coupled with water channel formation, creates ideal conditions for secondary colonization (Breslawec et al. 2021). The polysaccharide's conservation across phylogenetically distant genera suggests it could be a biofilm substrate that supports *Listeria* attachment and growth regardless of the primary biofilm former.

This passive colonization model finds strong support in recent experimental evidence. Voglauer et al. (2025) demonstrated that *L. monocytogenes* integrates into established multispecies biofilms within 2 h and resides within them for at least 7 days without significantly altering biofilm community composition or matrix components (eDNA, carbohydrates, and proteins). Crucially, this integration occurs efficiently at refrigeration temperatures (10°C), where *L. monocytogenes* persistence poses the greatest challenge to food safety.

Beyond passive integration, *L. monocytogenes* possesses diverse enzymatic capabilities that could actively modify biofilm matrices. The pathogen's documented ability to colonize *Pseudomonas fluorescens* biofilms and induce matrix overproduction suggests more than simple physical colonization (Puga et al. 2018). *L. monocytogenes* produces annotated chitinases ChiA and ChiB that hydrolyze β‐1,4‐linked chitin polymers (Chaudhuri et al. 2010), but enzyme annotation often underestimates substrate promiscuity. Many bacterial glycoside hydrolases demonstrate broader specificity than their names suggest, particularly for structurally related *N*‐acetylglucosamine polymers (Tawfik 2010). Additionally, *L. monocytogenes* encodes the glycoside hydrolase PssZ, which disrupts biofilm aggregates by hydrolyzing *N*‐acetylmannosamine‐galactose‐rich exopolysaccharides, demonstrating that *Listeria* has evolved enzymes capable of modifying complex polysaccharide matrices (Köseoğlu et al. 2015).

Significantly, *L. monocytogenes* genomes contain numerous hypothetical proteins of unknown function, many acquired through horizontal gene transfer events (Roy et al. 2018; Janež et al. 2021; Puente et al. 2025; Daeschel et al. 2022). Given that specialized PNAG‐hydrolyzing enzymes like Dispersin B exist in other bacteria, *L. monocytogenes* may harbor uncharacterized enzymes with similar capabilities (Breslawec et al. 2021). The pathogen's surface proteome includes multiple proteins with domains characteristic of carbohydrate‐binding or modification functions, suggesting a sophisticated toolkit for interacting with biofilm matrices (Janež et al. 2021). This enzymatic diversity, combined with passive colonization mechanisms, provides *L. monocytogenes* with multiple strategies for exploiting PNAG‐rich biofilm environments.

#### 3.5.3 Cross‐Feeding Opportunities

A review of the metabolic profiles of *Listeria*‐associated genera highlights multiple potential pathways for cross‐feeding relationships that may benefit *L. monocytogenes* in FPEs. Building on the biofilm matrix compatibility discussed above, these interactions extend beyond structural support to include the provision of growth factors, metabolic intermediates, and the creation of favorable microenvironmental conditions.

##### 3.5.3.1 Pseudomonas

Pseudomonas metabolic architecture creates conditions that correlate with enhanced L. monocytogenes survival in FPEs. The genus exhibits obligate Entner–Doudoroff pathway metabolism due to absent phosphofructokinase, generating glycerol‐3‐phosphate and pyruvate as overflow products (Nikel et al. 2021). *L*. *monocytogenes* possesses PTS systems capable of utilizing these compounds, though direct uptake in mixed biofilms requires further investigation (Sauer et al. 2019).

*Pseudomonas* demonstrates reverse diauxie metabolism, preferentially consuming organic acids over glucose when both are available (Berger et al. 2021). However, glucose availability is typically limited in FPEs, making organic acid metabolism and overflow product generation more relevant for potential cross‐feeding interactions. The pyruvate cycle generates acetate that could serve as a carbon source for *L*. *monocytogenes* during biofilm co‐residence.

The extensive *Pseudomonas* exometabolome includes riboflavin, which *L. monocytogenes* requires exogenously, and trehalose, which it efficiently catabolizes via PTS transport (Pinu and Villas‐Boas 2017). Temperature adaptation mechanisms, including cold shock proteins, enable *Pseudomonas* to maintain metabolic activity at refrigeration temperatures, sustaining cross‐feeding relationships during extended cold storage periods typical of FPEs.

Anecdotally, Rolon et al.’s shotgun sequencing data reveal that the dominant species identified in *L*. *monocytogenes*‐positive samples (*n* = 3) (*P. fluorescens*, *P. rhizospharae*, *P. koreensis*, *P. putida*, *P. syringae*, *P. chlororaphis*, and *P. resinovorans*) all maintain robust glycerol metabolism capabilities through multiple parallel pathways (Nikel et al. 2021; Poblete‐Castro et al. 2020). Notably, glycerol serves as the only carbon source supporting anaerobic rhamnolipid production in *P. aeruginosa*, indicating specialized metabolic processes under oxygen‐limited conditions typical of biofilm interiors (Xu et al. 2021). This anaerobic metabolism could create glycerol‐rich microniches accessible to *L. monocytogenes* during biofilm colonization.

##### 3.5.3.2 Psychrobacter

*Psychrobacter* species exhibit metabolic specialization that minimizes direct competition with *L. monocytogenes* while potentially providing beneficial compounds. These psychrotolerant bacteria prefer organic amino acids as carbon sources and generally don't break down polysaccharides or proteins (Bowman 2006; Denner et al. 2001). Notably, they can utilize uric acid as their sole carbon, nitrogen, and energy source (Bowman et al. 1996), representing a metabolic niche distinct from *L. monocytogenes* preferences.

The cryoprotectant production characteristic of *Psychrobacter* biofilms creates unique cross‐feeding opportunities. These bacteria actively secrete trehalose and glycerol into biofilm matrices as protection against freeze‐thaw cycles (Bowman 2006). Since *L. monocytogenes* possesses efficient trehalose and glycerol catabolic systems (Sauer et al. 2019), these cryoprotectants could serve as readily accessible carbon sources during cold stress conditions common in FPEs. This metabolic specialization suggests minimal competition with *L. monocytogenes* for preferred carbon sources while potentially providing nitrogen compounds beneficial to *Listeria* growth through amino acid catabolism.

##### 3.5.3.3 Acinetobacter

*Acinetobacter* is a diverse genus of organisms that has a wide range of metabolic capabilities and is found in a variety of environments, including water, plants, and soil (Adewoyin and Okoh 2018; Dandachi et al. 2019). A meta‐analysis of bacterial communities in FPEs found *Acinetobacter* spp. were among the most frequently detected regardless of commodity or nutrient levels, with the highest relative abundances found in meat and seafood processing facilities (Xu et al. 2023). Clade I is most prevalent in soil and habitats where people are present and seems to be rapidly adapting to human‐associated environments (Dahal et al., 2023). Clade II is most often linked with aquatic habitats, and Clade III with organic‐rich aquatic habitats and marine sediments (Dahal et al. 2023).

*Acinetobacter* metabolism reveals particularly intriguing potential synergies with *L. monocytogenes*. During sessile growth, *Acinetobacter* isolates from mushroom processing environments exhibited preferential glycerol utilization even in glucose presence (Lake et al. 2023), demonstrating metabolic preferences that could complement *L. monocytogenes* carbon utilization patterns. There are dual glycerol catabolism pathways in *Acinetobacter* species. Both the glycerol kinase and glycerol dehydrogenase routes generate significant glycerol‐3‐phosphate flux (Pirog et al. 2012). Since *Listeria sensu stricto* can produce glycerol via exogenous enzymatic activity, particularly through phospholipase‐mediated membrane degradation, *Acinetobacter* could effectively process this overflow glycerol production.

A particularly significant finding is that *Acinetobacter baumannii* biofilm formation is inhibited by exogenous riboflavin, trehalose, or sorbitol (Laal‐Kargar et al. 2020; Gedefie et al. 2021). Notably, *L. monocytogenes* requires exogenous riboflavin and actively catabolizes trehalose and sorbitol via its conserved PTS systems (Sauer et al. 2019). This creates a scenario where *L. monocytogenes* could enhance *A. baumannii* biofilm formation through normal metabolic processes by removing compounds that would otherwise inhibit biofilm development.

##### 3.5.3.4 Veillonella

Among all the microbial genera reportedly associated with *L. monocytogenes*, *Veillonella* stands apart for the methodological rigor with which its association was established. While multiple studies have identified *Pseudomonas*, *Psychrobacter*, and *Acinetobacter* as positively correlated with *Listeria* presence, the Shedleur‐Bourguignon et al. (2021) study employed a uniquely robust statistical approach that strengthens confidence in microbial associations.

Shedleur‐Bourguignon et al. (2021) properly controlled for autocorrelations introduced by repeated measurements both longitudinally and by sampling location. The results may have highlighted the importance of appropriate statistical controls; despite *Pseudomonas*, *Psychrobacter*, and *Acinetobacter* being among the dominant taxa in their pork processing environment, only *Veillonella* showed a significant association with *L. monocytogenes* when these controls were applied. This methodological distinction reveals a sobering possibility about the broader literature. Without controlling for repeated measures, samples from the same surface at different timepoints are treated as independent, potentially inflating the significance of abundant taxa that appear in multiple *Listeria*‐positive samples. The consistent identification of the same aerobic genera across studies may reflect their environmental dominance rather than specific ecological relationships with *L. monocytogenes*.

*Veillonella*'s emergence from this methodological filter is therefore significant. It represents one of the few statistically robust microbial associations with *L. monocytogenes* identified to date, and its biological characteristics support this distinction. *Veillonella* spp. are well‐established bridging organisms in biofilm ecology, particularly known for their role in oral biofilms, where they facilitate transitions between aerobic and anaerobic metabolism (Zhou et al. 2021). This bridging function provides compelling support for the secondary colonizer hypothesis—that *L. monocytogenes* exploits niches created by other organisms rather than simply co‐occurring with environmental dominants.

The sophistication of the *Veillonella*‐*Listeria* relationship becomes apparent when examining their shared metabolic capabilities. *Veillonella* is among the few genera that possess bacterial microcompartments (BMCs) and extracellular electron transfer (EET) systems similar to those found in *L. monocytogenes* (Light et al. 2019; Zeng et al. 2021; Zeng et al. 2019). Both organisms can utilize specialized substrates through these systems and engage in anaerobic respiration using external electron acceptors. However, transcriptomic analysis revealed a remarkable pattern: when *Veillonella gordonii* grows in multispecies biofilms compared to monoculture, it has been reported to demonstrate an extraordinary 840‐fold downregulation of fumarate reductase expression (Mutha et al. 2019).

This metabolic withdrawal is extraordinary. *Veillonella* essentially cedes the anaerobic respiration territory to *L. monocytogenes*, eliminating competition for the very processes that mounting evidence suggests are critical to *L. monocytogenes* persistence through its cobalamin‐dependent gene cluster (CDGC). Multiple studies have documented CDGC upregulation when *L. monocytogenes* encounters other bacteria: co‐culture with *Psychrobacter* spp. Increased CDGC transcription (Anast and Schmitz‐Esser 2020), persistent strains showed higher CDGC expression than sporadic strains (Fox et al. 2011), co‐culture with *Carnobacterium pisciola* triggered CDGC upregulation (Nilsson et al. 2005), and competitive stress with *Bacillus subtilis* caused CDGC upregulation (Tirumalai 2015). Chen et al. (2021) found similar CDGC transcriptional enhancement with *Ralstonia* spp., while biofilm formation itself triggers CDGC upregulation within 24 h (Gray et al. 2021).

*Veillonella* spp.'s potential metabolic cooperation extends beyond competitive withdrawal. These anaerobes have high tolerance to oxygenic conditions (Zhou et al. 2017), positioning them perfectly at aerobic–anaerobic interfaces within biofilms. They metabolize lactate—a common overflow product from aerobic biofilm colonizers—converting it to acetate, propionate, CO_2_, and H_2_ (Zhou et al. 2021). This removes potentially inhibitory organic acids while generating short‐chain fatty acids that may benefit *L. monocytogenes*. Additionally, *Veillonella* species convert nitrate to nitrite and subsequently to ammonia (Zhou et al. 2021), removing bactericidal compounds to which *L. monocytogenes* is susceptible (Majou and Christieans 2018). *V. gordonii* even downregulates glutamate assimilation genes in co‐culture biofilms (Zhou et al. 2021), potentially increasing nitrogen availability since glutamate can seamlessly substitute for ammonia as the primary source of nitrogen in *L. monocytogenes* metabolism (Kutzner et al. 2016; Sauer et al. 2019).

#### 3.5.4 Competitive Interactions: Balancing Antagonism and Cooperation

While many of the identified relationships suggest cooperation, competitive interactions may also shape *L. monocytogenes* persistence in FPEs. Understanding this balance of antagonism and cooperation provides further insights into *Listeria's* ecological strategy. Interspecific interactions within *Pseudomonas* vary significantly at the species and strain level. Townsend et al. (2023) found an increased relative abundance of different *Pseudomonas* E ASVs in both *Listeria* spp.‐positive and ‐negative samples, illustrating nuanced interactions based on environmental context.

Laboratory data agree with the environmental data in that different species or strains of *Pseudomonas* interact with *Listeria* spp. differently (Lalucat et al., 2020). For instance, an in vitro study found *L*. *monocytogenes* greatly increased *P*. *putida*’s tolerance to exposure to sublethal levels of quaternary ammonia compounds (QAC) (Giaouris et al. 2013), while another showed that preformed *P*. *putida* biofilms increased attachment of *L*. *monocytogenes* on stainless steel surfaces (Hassan et al., 2004). Recently, Lake et al. (2023) found higher *L*. *monocytogenes* counts after co‐culture with *P*. *fragi* when compared to monoculture. However, an older study found that biofilm formation by *L*. *monocytogenes* was reduced in coculture with *P*. *fragi* (Norwood and Gilmour 2000). Yet another group of researchers discovered that *L*. *monocytogenes* is also able to colonize the depths of preformed *P*. *fluorescens* biofilms while inducing them to secrete additional EPS and promoting their dispersal (Puga et al. 2018), contradicting another study that found that biofilm development was inhibited when *L*. *monocytogenes* was co‐cultured with *P*. *fluorescens* (Kragh and Truelstrup Hansen 2019).

The negative correlation between *Acinetobacter* and *L. monocytogenes* observed in tree fruit facilities by Tan et al. (2019) contrasts with the positive associations found in meat processing environments (Belk et al. 2022; Zwirzitz et al. 2021). This discrepancy may reflect context‐dependent competition or cooperation based on specific nutrient availability or environmental conditions.

Notably absent from the list of *Listeria*‐associated genera were common FPE aerobic biofilm‐formers like *Bacillus*, *Staphylococcus*, and *Streptococcus* (Xu et al. 2023). These genera contain species known for their production of antimicrobial compounds, including bacteriocins that may inhibit *L. monocytogenes* growth. This pattern of associations and non‐associations suggests that *L. monocytogenes* persistence in FPEs may depend on a balance between cooperative partners that provide structural support and metabolic benefits while avoiding strongly antagonistic species. The consistent presence of aerobic biofilm formers in *Listeria*‐positive samples, coupled with the notable absence of known antagonistic species, indicates a sophisticated ecological strategy that leverages specific microbial partnerships while evading competitive exclusion.

## 4 Conclusion

This systematic review and meta‐analysis represent the first comprehensive examination of culture‐independent metagenomic studies investigating *L*. *monocytogenes* persistence in FPEs. The meta‐analysis of six studies encompassing 1659 environmental samples revealed no significant correlation between *Listeria* presence and overall microbial community alpha diversity (Shannon: *z* = −0.89, *p* = 0.40). This finding challenges previous assumptions about the relationship between microbial diversity and pathogen persistence, suggesting that *L*. *monocytogenes* survival depends on specific ecological partnerships rather than community‐wide characteristics.

Differential abundance analyses identified three genera most frequently associated with *Listeria* presence across multiple studies: *Pseudomonas* (four of seven studies), *Psychrobacter* (three of seven studies), and *Acinetobacter* (three of seven studies). These Gammaproteobacteria are characterized as aerobic biofilm formers capable of growth at refrigeration temperatures. One study using rigorous mixed‐effects modeling identified *Veillonella* as significantly associated with *L*. *monocytogenes* presence, demonstrating extraordinary metabolic cooperation through 840‐fold downregulation of fumarate reductase expression in multispecies biofilms. This metabolic withdrawal effectively cedes anaerobic respiration territory to *L*. *monocytogenes* while creating favorable environmental conditions through lactate metabolism and nitrate reduction.

Analysis of biofilm matrix composition reveals that PNAG production may represent a unifying feature across *Listeria*‐associated genera. This polysaccharide creates structured matrices with water channels that facilitate rapid *L*. *monocytogenes* colonization. Recent experimental validation demonstrates that *L*. *monocytogenes* integrates into established multispecies biofilms within 2 h without altering community composition, supporting a passive colonization strategy that may explain persistence despite routine sanitation efforts.

These findings indicate that *L*. *monocytogenes* persistence appears to be mediated by specific microbial partnerships that provide structural protection and metabolic benefits rather than by overall community diversity metrics. Understanding these ecological relationships may inform the development of targeted control strategies that focus on disrupting biofilm‐forming genera and their supportive functions. Future research applications may include PNAG‐targeting enzymatic cleaners, environmental modifications to limit cross‐feeding substrates, and promotion of competitive exclusion organisms that were notably absent from *Listeria*‐positive samples. The identification of specific microbial partnerships and their underlying mechanisms provides a foundation for developing precision interventions that target the ecological conditions enabling *L*. *monocytogenes* persistence rather than relying solely on broad‐spectrum sanitation approaches.

**Nomenclature**
ASV: amplicon sequence variant
Bap: biofilm‐associated protein
BMC: bacterial microcompartment
CDGC: cobalamin‐dependent gene cluster
c‐di‐GMP: cyclic di‐GMP
CV: crystal violet
EA: ethanolamine
eDNA: extracellular DNA
EET: extracellular electron transfer
EFSA: European Food Safety Authority
EPS: extracellular polymeric substances
FCS: food contact surface
FPE: food processing environment
Fri: ferritin‐like protein
FSTA: Food Science and Technology Abstracts
INP: ice‐nucleating protein
NFCS: nonfood contact surface
PD: 1,2‐propandiol (propylene glycol)
PERMANOVA: permutational analysis of variance
PNAG: Poly‐β‐^1,6^‐*N*‐acetyl glucosamine
PRISMA: Preferred Reporting Items for Systematic reviews and Meta‐Analyses
PTS: phosphotransferase system
QAC: quaternary ammonium compound
ROSES: Reporting Standards for Systematic Evidence Syntheses
RTE: ready‐to‐eat
SLR: systematic literature review
SME: subject matter expert
spp.: species

## Author Contributions

**Jack Burnett**: conceptualization, investigation, writing–original draft, methodology, formal analysis, writing–review and editing, data curation, visualization, software. **David Buckley**: conceptualization, funding acquisition, methodology, supervision, writing–review and editing, project administration, visualization. **Dale A. Grinstead**: conceptualization, writing–review and editing, validation. **Haley F. Oliver**: project administration, conceptualization, writing–review and editing, resources, funding acquisition.

## Conflicts of Interest

Dr. Jack Burnett was both a graduate research assistant at Purdue University and an employee of Diversey, Inc., at the time this research was conducted. Diversey, Inc. funded Dr. Burnett's doctoral research. The other authors declare no conflicts of interest.

## Supporting information




**Supporting Table 1**: crf370277‐sup‐0001‐Table1.xlsx

**Supporting Table 2**: crf370277‐sup‐0002‐Table2.xlsx

**Supporting Table 3**: crf370277‐sup‐0003‐Table3.xlsx

**Supporting Table 4**: crf370277‐sup‐0004‐Table4.xlsx

**Supporting Table 5**: crf370277‐sup‐0004‐Table4.xlsx
